# The Effect of Walnut Flour on the Physical and Sensory Characteristics of Wheat Bread

**DOI:** 10.1155/2019/5676205

**Published:** 2019-01-20

**Authors:** Noha M. Almoraie

**Affiliations:** Food and Nutrition Department, Faculty of Home Economics, King Abdulaziz University, Jeddah, Saudi Arabia

## Abstract

The study was carried out to demonstrate the effect of walnut flour enhancement on the physical, nutritional, and sensory quality of bread. Walnut flour was prepared by soaking, deshelling, oven drying, and sieving whole walnuts. The wheat flour was supplemented with walnut flour by 0, 20, 30, 40, and 50% of the total amount. Standard procedures were taken to estimate the proximate composition of wheat and walnut flour and bread samples. A comparison between the control and supplemented bread was made, where the physical characteristics (weight, volume, and specific volume) and sensory quality were checked. The enhanced bread, where the percentage was between 20 and 50%, appeared to have a significant increase in protein, fat, linoleic acid, and *α*-linolenic acid and a decrease in carbohydrate and fibre values. Increased walnut flour replacement showed that physical properties, loaf volume and specific loaf volume, have declined. The sensory attributes between the unsupplemented and supplemented bread showed major differences. As an outcome, substituting 30% walnut flour gave the best overall quality of bread acceptability.

## 1. Introduction

Usage of non-traditional components establishes new properties of wheat flour and could be achieved by milling product from walnuts. Relating to these components, they also develop greater nutritional benefits of products in regard to the protein content and other beneficial ingredients for health. Incidentally, the nutritional benefits were improved by adding these components. Walnuts, compared to wheat, have a definite chemical structure with greater nutritional benefits; therefore, they are highly recommended. Despite the improvement of the analytic features, composites have increased bread quality and nutritional benefits. They are related to enhanced blood lipoprotein profile, antioxidant activity, antiatherogenic impact, decrease of tumour initiation or advancement, repair of DNA damage, regulation of cell differentiation and proliferation control of immunological activity, induction of phase II metabolic enzymes, and inflammatory reaction [1, 2]. Walnuts are an intriguing source of vegetable protein having 20% of extremely digestible protein and a fair composition of fundamental amino acids [3]. These nuts contain about 50% to 70% oil rich in polyunsaturated fatty acids (around 70% total fatty acids), in particular linolenic acid [4]. Being rich in magnesium and a great source of tocopherols, containing four forms of tocopherol (*α*, *β*, *γ*, and *δ*), sterols, carotenoids, and aliphatic alcohols [5], walnuts also contain a battery of phytochemicals, with antioxidant properties such as phenolic compounds [6]. The helpful impact of this extraordinary fatty acid profile has been a point of discussion for many studies. Band and Hu deduced in 2009 that the short-term studies have promising results for walnut enhanced regimens; however, long-term studies are necessary to show better outcomes [7]. Raw walnuts contain glyceryltriacylates of the omega-3 fatty acid alpha-linolenic acid, which is inadequate in human as long-chain omega-3 fatty acids and antioxidants [8]. A study suggested the eating walnuts might enhance the utilization of body fat in overweight adults, because the intake of walnuts does not affect the total consumption as the fat oxidation increases while the carbohydrate oxidation decreases [9]. A decrease in endothelial dysfunction in correlation to a high fat meal is an effect of walnuts [10]. Walnuts are valuable source of omega 3 fatty acids such as linoleic acid, alpha-linolenic acid, and arachidonic acids in monounsaturated fatty acids (about 72%) such as oleic acid. The typical consumption of walnuts within the diet supports lower total cholesterol and LDL and increases HDL levels in the blood. Research studies advocate that a Mediterranean diet helps to avoid coronary artery disease and strokes since the diet is rich in monounsaturated fatty acids and omega-3 fatty acids that favour healthy blood lipid [11]. Also, other studies implied that the character of omega-3 fatty acids being anti-inflammatory helps to minimize the risk of blood pressure, coronary artery disease, strokes, and breast, colon, and prostate cancers [12, 13]. A noteworthy issue confronting developed countries is malnutrition which assisted in children mortality, poor physical, low resistance to diseases and intellectual development. Another issue is the expense on wheat imports and supplementation of walnut flour adds to the wheat flour by increasing the protein content and other nutrients. Therefore, the objective of the present study was to determine the possibilities of using walnuts and its impact on the physical and sensory characteristics on wheat bread.

## 2. Materials and Methods

### 2.1. Materials

Wheat flour (75% extraction) was obtained from grain silos and flour mills organization, Jeddah, Saudi Arabia. Walnuts, sugar, and salt were purchased from local markets in Jeddah, Saudi Arabia. All chemicals used are of analytical grade.

### 2.2. Preparation of Walnuts

The whole walnuts (*Juglans regia*) were initially washed completely to get rid of any adhering contaminants. They were then cooked for half an hour in an exceeding steel pot to get rid of the shells. With the help of a stainless-steel knife, the deshelled walnuts were reduced into smaller sizes. Then they were blanched in a warm water for five minutes before debilitating. Once blanched, the walnuts were dried in an exceedingly hot air kitchen appliance at 60°C for five hours to get rid of moisture. They were then processed and sieved to supply the walnut flour [14].

### 2.3. Preparation of Dough Samples

Different composite flour samples were prepared by combining 100%, 80%, 70%, 60%, and 50% of wheat flour and 0%, 20%, 30%, 40%, and 50% of walnuts flour, respectively (Table 1). Wheat flour was coded with A and Walnuts flour with B according to [15].

### 2.4. Proximate Composition

The approximate composition of bread samples was determined following the official standard method [16]. Moisture content was measured using oven drying method and weight measurements before and after drying (AOAC 935.29). Protein was determined by Kjeldahl method (AOAC 988.05). Oil and ash were estimated by Soxhlet method (AOAC 963.15) and drying methods (AOAC 942.05), respectively. Carbohydrate was calculated by differences. Fatty acids composition of raw materials and bread was performed using a gas chromatography. Fatty acid methyl esters were prepared in accordance with European Standard [17]. The fatty acids were identified using standard lipid purchased from Sigma Aldrich.

### 2.5. Determination of Dough Rheology

The rheological properties of wheat dough and with different concentrations of walnuts flour (20, 30, 40, and 50%) followed the standard methods of American Association of Cereal Chemists [18]. Farinogragh and amylograph measurements were carried out according to AACC54-21 and 54-10 methods, respectively, using Barbender instruments (C.W. Brabender Instrument Inc., South Hackensack, NJ, USA).

### 2.6. Physical Characteristics of Bread

Physical characteristics of bread samples such as loaf weight, loaf volume, and specific loaf volume were evaluated. Loaf weight was measured using a laboratory scale (CE- 410I, Camry Emperors, China) after half an hour when the loaves were removed from the oven and the readings recorded in grams. Loaf volume was measured as modified by Giami et al. (2004) [19] using the rapeseed displacement method. The specific loaf volume was determined as described by Araki et al. (2009) [20], and three measurements were taken from every sample. The specific loaf volume = the loaf volume/ loaf weight (cm^3^/g)

### 2.7. Sensory Attributes

Sensory evaluation of bread samples was performed 2 h after baking by 50 females selected among students, staff, and faculty members of King Abdelaziz University, Jeddah, Saudi Arabia. The sensory quality of bread was assessed using a 5-point hedonic scale (5 = like, 1 = dislike) used for the external and internal properties (colour, texture, taste, flavor, and overall acceptability) of the bread. The bread samples were coded with random numbers of three digits and served randomly to the panelists. Sensory analysis was carried out in three sessions, and the mean values of the scores of 50 evaluators were calculated and used in data analysis for each sample and session.

### 2.8. Statistical Analysis

Data were analysed using SPSS Inc., version 25, Chicago, IL, USA. Data were presented as means and standard deviation (SD). All data were subjected to variance analysis (ANOVA) and the results were compared using Turkey's test and the statistical significance meaning was defined as* p *≤ 0.05.

## 3. Results and Discussion

### 3.1. Proximate Composition of Flours Samples

The proximate composition of wheat flour and walnut flour and their composition are given in Table 2.

In general, the chemical quality of the combined bread was affected by the supplementation of wheat flour with walnut flour. Raising the percentage of walnut supplementation increases the values for protein, ash, fat, fibre, linoleic acid (omega 6 fatty acid), and *α*-linolenic acid (omega 3 fatty acid) except for carbohydrate contents. The moisture values for the flours were 8.20%, 5.80%, 6.24%, 9.57%, and 7.15%, respectively. It seems to be within the usual moisture values of dried food (no more than 9%, for flour blends). The five samples showed distinctions in the moisture content. There was a good indication that the low moisture of these samples determined a long shelf life. Materials that contain more than 12% moisture are considered to have essentially flour and starch and less storage stability than those with lower moisture content. Consequently, water content of 10% is commonly specified for flours and other related products. The sample AB3 (60% wheat flour-40% walnut flour) had a significantly higher moisture content that differed from other samples. Various food materials have different moisture retention capabilities that can exist as occluded or absorbed water [21–23]. The addition of walnut to the wheat flour influenced the binding of moisture; as a result, the composite flour samples had a reduced moisture content as seen in Table 2. Bread samples with walnut flour supplementation showed an increase in the protein content in the range of 17.60 to 25.10% in comparison to wheat flour (12.75%), due to the fact that the wheat flour has lower protein content than walnut flour. The fat content in the walnut flour supplementation also displayed a raise with a range of 28.83- 37.25% in composite bread samples. It could be associated with the high percentage of polyunsaturated fatty acids where the results showed that the percentages of omega 3 and omega 6 were very high compared with the control. Thus, the addition of walnuts flour to bread had a positive effect on nutritional values of the product. The samples had significantly higher (*p* ≤ 0.05) content of linoleic acid and *α*-linolenic acid than control bread. The high content of omega 3 fatty acids in the diet provides many health benefits. It is essential for proper brain functions and has beneficial effects in cardiovascular diseases and in prevention of arthritis [24]. For that reason, incorporating higher levels of walnut flour in the bread derived an enhancement in protein and fat content considering lowering the amount of wheat flour. On the contrary, carbohydrate content was reduced upon the supplementation of wheat flour with walnut flour, where the carbohydrate content was highest in the control (68.21%) and lowest in sample containing 50% walnuts flour (17.95%). It was implied that the main contributor to the carbohydrate content was wheat flour. The composite bread sample consisted of energy values in the range of 270 to 299 Kcal. This could refer to the fact that the higher energy values of the 50% walnut bread are due to higher fat content compared to other bread samples.

### 3.2. Dough Rheology

The dough rheological properties of wheat flour and supplementation of wheat flour with walnuts flour were analysed by farinograph and amylograph. The farinograph results indicated that supplementation of wheat flour with walnuts flour significantly affected the water absorption, dough stability, and degree of softening (Table 3). Increasing the supplementation of walnuts flour in the dough progressively (*p* ≤ 0.05) increased the water absorption from 60% in control to 65% in wheat flour supplemented with 50% walnuts flour. This could be due to the increase of the protein solubility and content of the dough following the addition of walnuts whose protein is characterized by its high solubility and absorption capacity. Furthermore, added walnuts could result in a structural modification in the dough which may allow absorption of more water due to hydrogen bonding. High water absorption capacity of dough represents consistency which is one of the appealing characteristics in bread making. In addition, several research reports have demonstrated that supplementation of wheat flour with vegetable and legumes flour, dairy products, or protein isolates significantly increased the water absorption capacity [25–28]. This study also showed that incorporation of walnuts flour in wheat dough decreased the dough stability compared to control and the reduction was concomitant with an increase in the concentration of walnuts in the dough. The reduction could be due to the fact that added walnuts constituents could disrupt the wheat gluten-starch network, competing with wheat flour proteins for water, and then decrease its stability. A similar decrease in dough stability was also observed by Sabanis and Tzia [29], Anton et al. [30], Gadallah et al. [31], and Pasha et al. [32] as the percentage level of legume flour in the blend increased. Our results also revealed that the effects of walnuts fortification on the degree of softening were minor. Overall, the farinograph analysis demonstrated that incorporation of different supplementation of walnuts in bread formulations positively affected the water absorption capacity whereas it showed an adverse impact on the dough stability. The amylograph results (Table 3) showed that fortification of wheat flour with walnuts flour significantly (*p* ≤ 0.05) affected the gelatinization temperature, peak viscosity, and temperature at peak viscosity of fortified dough compared to control samples. Gelatinization temperature increased with increase the walnuts flour in the dough, while the peak viscosity and temperature at peak viscosity showed concomitant reduction as the walnuts flour increased. Our finding agreed with other reports which have indicated that addition of legumes protein isolates reduced the peak viscosity of the dough [33, 34]. In contrast, other studies showed that increases in peak viscosity and gelatinization temperature were observed in composite flours of wheat, cereals, legumes, or sago flours [35–38]. The difference could be attributed to the variation in the added materials: in the latter, protein isolates are devoid of sugars or starches whereas, in the former, the whole materials are added which might contain sugars and starches that increase the viscosity and gelatinization temperature.

### 3.3. Physical Characteristics of Bread Samples

Outcomes of the physical attributes of composite bread samples incorporating various percentages of walnut flour supplementation in comparison to the control are given in Table 4. The weight of the entire samples of walnut flour bread loaves were greater than the control wheat bread. Nonetheless, the higher the level of supplementation of walnut flour the lower the loaf volume and specific volume. The lowest loaf volume (645.9±0.41 cm^3^) and specific volume (2.63±0.06 cm^3^/g) were observed at 50% walnuts flour supplementation. The increased weight and dense texture of the composite bread samples is developed by less retention of carbon dioxide in the mixed batter, whereas the decrease in the volume and specific volume of the composite loaf is due to the dilution effects on gluten with addition of walnuts flour to the wheat flour. When fermenting, the carbon dioxide produced by the yeast is being expanded and trapped in the dough causing elasticity by the gluten fraction. At the point when gluten coagulates and is affected by the heat during baking, it delivers as the framework of the loaf, which turns generally rigid and does not collapse. Furthermore, adding walnut flour causes the increase in fibre content of the composite flour which may have noticeable effects on dough properties yielding for higher water absorption, giving persistence and endurance, and smaller extensibility as to those taken without fibre supplementation [39]. Likewise, the unfavourable effects of fibre supplementation on dough structure and loaf volume have implied to be caused by diluting the gluten network, and that will generate a reduction in gas retention in place of gas production [39]. Consequently, the results of the current study suggest that the appropriate percentage of supplementation of walnut flour is 20%, ensuring the most convenient weight and volume attributes for the bread, to be compared with the wheat bread. Enhancing wheat flour with more than 20% leads to a decrease in weight and volume; oilseed flours, protein, and legume concentrates have been reported [40–42].

### 3.4. Sensory Evaluation of Bread

**Figure 1:**
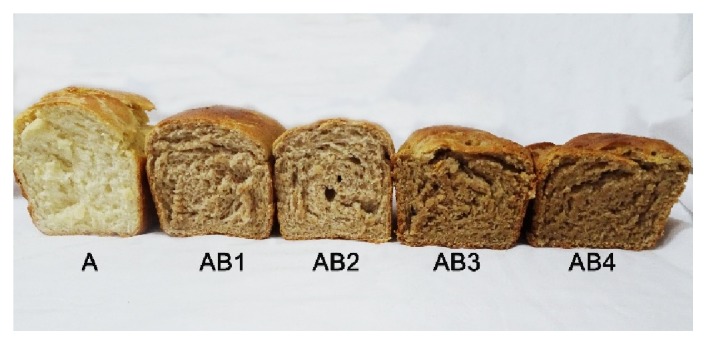
It shows the sensory evaluation of bread. Wheat flour 100% (A), wheat flour 80% and walnut flour 20% (AB1), wheat flour 70% and walnut flour 30% (AB2), wheat flour 60% and walnut flour 40% (AB3), and wheat flour 50% and walnut flour 50% (AB4).

Sensory assessment is a vital measure for quality assessment in a newly developed food product to attract consumers and to meet their requirements [43]. The choice of a food product depends on various aspects like character, mood, and experience and characteristics such as sensory properties, health and nutrition, and price and value [44].  Table 5 shows the sensory attributes of composite bread of the different percentages of walnut flour supplementation added to the wheat flour. A noteworthy difference is gathered by the evaluation of the crumb colour between the composite bread samples and the 100% wheat bread. The results clearly show that bread prepared from 100% wheat flour had highest score (4.67) followed by bread prepared from 80% of wheat flour and 20% walnut flour (4.65). Breads prepared from 70:30, 60:40, and 50:50 wheat flour to walnut flour combinations were fairly rated by assessors with respect to crumb colour. There is an increase in intensity of crumb colour with higher level of supplementation. Darkness may be attributed to the colour of walnut as shown in Figure 1. On the other hand, inclusion of 40% and 50% of walnut flour significantly reduced the crumb texture compared to control and that supplemented with 20% and 30% of walnut flour. The results for crumb texture revealed that the bread made with 70% wheat flour and 30% of walnut flour had the highest scoring value (4.69) whereas bread with 50% walnut flour had the lowest value (2.80). The addition of walnuts flour caused extra fat and moisture of the bread crumb, but it was generally not negatively evaluated by the panelists. Only for AB4 samples the significantly lower notes for crumb texture were evaluated in comparison to control bread. With the evaluation for the taste and flavour of bread, the quality score ranged from 3.55 to 4.80. Scoring 4.80, 30% walnut flour was the highest value followed by bread prepared from 20% walnut flour. A reason for this may be caused by the bitter taste of some inherent walnut flour compounds, especially at high temperatures, as reported by Santos et al. (2017) [45]. There are factors that contribute to the final product of bread including quantities of water absorbed during dough mixing; the baking conditions (time variables and temperature); the states of the bread components, such as starch, fibre, and protein whether damaged or undamaged [46]. The overall evaluation also shows that degree of supplementation influences the overall approval of the bread samples. Bread made from 30% walnut flour and 70% of wheat flour had maximum score with 4.74 compared to control (4.66) and other walnut flour supplementation. In particular, the baking properties of composite flour are usually defective in addition to the organoleptic characteristics of the products, because of the dilution of the gluten content [47]. Overall our findings demonstrated that supplementation of bread formula with walnut flour at 30% with 70% wheat flour is recommended for improving the nutritional and sensorial attributes of bread.

## 4. Conclusions

The current study was to determine the possibilities of using walnuts in the production of the wheat bread and determine the impact of walnuts on physical and sensory characteristics of wheat bread. Bread samples that were enhanced with walnut flour were found to be highly nutritional (higher protein, fat, and fatty acids) compared to 100% wheat bread. Nevertheless, walnut supplemented wheat flour was substantially affected by rheological properties (water absorption capacity, dough development, gelatinization temperature, and peak viscosity), physical properties (bread weight, volume, and specific volume), and sensory quality of bread. Supplementation of 30% walnut flour into wheat flour resulted in sensory acceptability that was best for the bread.

## Figures and Tables

**Table 1:** Bread making method.

Ingredients	A	AB1	AB2	AB3	AB4
Wheat flour	100%	80%	70%	60%	50%
Walnuts flour	0	20%	30%	40%	50%
Salt	2%				
Sugar	5%				
Instant yeast	3%				
Water*∗*	50%				
Dough mixing time/mixing speed	4 min/235 rpm				
Dough fermentation conditions	30°C, RH 75%, 90 min (transfixion after 60 min)				
Dough proofing conditions	30°C, RH 75%, 60 min				
Baking conditions	230°C, 30 min				

*∗* Added water calculated based on the farinograph results of formulated dough. wheat flour (A) and walnut flour (B) used in composite flour formulation. RH, relative humidity.

**Table 2:** Proximate composition of wheat and walnut flour samples.

Parameters	A	AB1	AB2	AB3	AB4
Moisture (%)	8.20^d^ ± 0.04	5.80^a^ ± 0.11	6.24^b^ ± 0.06	9.57^e^ ± 0.05	7.15^c^ ± 0.03
Protein (%)	12.75^a^ ± 0.01	17.60^b^ ± 0.02	18.55^c^ ± 0.03	21.27^d^ ± 0.20	25.10^e^ ± 0.01
Fibre (%)	1.27^e^ ± 0.06	3.81^a^ ± 0.40	4.26^b^ ± 0.30	5.17^c^ ± 0.01	9.13^d^ ± 0.10
Ash (%)	2.23^b^ ± 0.02	1.50^a^ ± 0.30	2.24^c^ ± 0.10	3.17^e^ ± 0.03	2.83^d^ ± 0.06
Fat (%)	2.51^a^ ± 0.06	28.83^b^ ± 0.40	31.14^c^ ± 0.30	35.47^d^ ± 0.01	37.25^e^ ± 0.10
Linoleic acid (%)	52.1^a^ ± 0.01	62.8^b^ ± 0.12	69.3^c^ ± 0.05	73.4^d^ ± 0.25	78.2^e^ ± 0.36
*α*-linolenic acid (%)	2.50^a^ ± 0.23	8.57^b^ ± 0.18	9.57^c^ ± 0.11	10.66^d^ ± 0.03	11.5^e^ ± 0.21
Carbohydrate (%)	68.21^e^ ± 0.01	45.50^d^ ± 0.12	36.04^c^ ± 0.06	24.17^b^ ± 0.10	17.95^a^ ± 0.12
Energy (kcal/100g)	275.2^b^ ± 0.03	270.1^a^ ± 0.10	276.4^c^ ± 0.05	287.5^d^ ± 0.05	299.5^e^ ± 0.50

Mean ± standard deviation; different letters in the same column represent significant differences (*p* ≤ 0.05 between samples). Wheat flour 100% (A), wheat flour 80% and walnut flour 20% (AB1), wheat flour 70% and walnut flour 30% (AB2), wheat flour 60% and walnut flour 40% (AB3), and wheat flour 50% and walnut flour 50% (AB4).

**Table 3:** Farinograph and Amylograph readings of wheat and walnut bread samples.

Characteristics	A	AB1	AB2	AB3	AB4
Water absorption (%)	61.0^a^ ± 0.11	63.0^b^ ± 0.23	64.0^c^ ± 0.11	64.0^c^ ± 0.11	65.0^d^ ± 0.21
Dough stability (min)	9.5^d^ ± 0.15	8.3^b^ ± 0.01	8.4^c^ ± 0.10	8.4^c^ ± 0.08	8.2^a^ ± 0.02
Degree of softening (BU)	23.0^c^ ± 0.12	24.0^d^ ± 0.18	22.0^b^ ± 0.14	21.0^a^ ± 0.12	21.0^a^ ± 0.15
Gelatinization temperature (°C)	60.2^a^ ± 0.43	62.1^b^ ± 0.38	63.0^c^ ± 0.23	63.1^d^ ± 0.11	63.2^e^ ± 0.18
Peak viscosity (BU)	670^e^ ± 0.21	580^d^ ± 0.25	478^c^ ± 0.34	416^b^ ± 0.12	401^a^ ± 0.11
Temperature at peak viscosity (°C)	88.1^e^ ± 0.12	86.2^d^ ± 0.09	83.9^c^ ± 0.16	82.5^b^ ± 0.10	80.9^a^ ± 0.21

Mean ± standard deviation; different letters in the same column represent significant differences (*p* ≤ 0.05 between samples). Wheat flour 100% (A), wheat flour 80% and walnut flour 20% (AB1), wheat flour 70% and walnut flour 30% (AB2), wheat flour 60% and walnut flour 40% (AB3), and wheat flour 50% and walnut flour 50% (AB4).

**Table 4:** Physical characteristic of wheat and walnut bread samples.

Characteristics	A	AB1	AB2	AB3	AB4
Weight (g)	219.4^a^ ± 0.1	232.2^b^ ± 0.3	237.9^c^ ± 0.1	240.4^d^ ± 0.4	245.2^e^ ± 0.2
Volume (cm^3^)	944.2^e^ ± 0.1	769.1^d^ ± 0.2	709.12^c^ ± 0.1	662.5^b^ ± 0.1	645.9^a^ ± 0.4
Specific volume (cm^3^/g)	4.3^e^ ± 0.2	3.31^d^ ± 0.5	2.98^c^ ± 0.2	2.75^b^ ± 0.5	2.63^a^ ± 0.6

Mean ± standard deviation; different letters in the same column represent significant differences (p ≤ 0.05 between samples). Wheat flour 100% (A), wheat flour 80% and walnut flour 20% (AB1), wheat flour 70% and walnut flour 30% (AB2), wheat flour 60% and walnut flour 40% (AB3), and wheat flour 50% and walnut flour 50% (AB4).

**Table 5:** Sensory evaluation of wheat and walnut bread samples.

Parameter	A	AB1	AB2	AB3	AB4
Crumb colour	4.67^e^ ± 0.21	4.65^d^ ± 0.13	4.56^c^ ± 0.11	4.28^b^ ± 0.24	3.23^a^ ± 0.42
Crumb texture	4.61^d^ ± 0.19	4.63^c^ ± 0.11	4.69^e^ ± 0.30	4.12^b^ ± 0.41	2.80^a^ ± 0.31
Taste and flavour	4.54^c^ ± 0.20	4.72^d^ ± 0.23	4.80^e^ ± 0.18	3.95^b^ ± 0.25	3.55^a^ ± 0.33
Overall acceptability	4.66^c^ ± 0.12	4.71^d^ ± 0.9	4.74^e^ ± 0.06	3.48^b^ ± 0.10	3.43^a^ ± 0.12

Mean ± standard deviation; different letters in the same column represent significant differences (*p* ≤ 0.05 between samples). Wheat flour 100% (A), wheat flour 80% and walnut flour 20% (AB1), wheat flour 70% and walnut flour 30% (AB2), wheat flour 60% and walnut flour 40% (AB3), and wheat flour 50% and walnut flour 50% (AB4).

## Data Availability

The quantitative data used to support the findings of this study are included within the article.
